# Constructions and experiences of motherhood in the context of an early intervention for Aboriginal mothers and their children: mother and healthcare worker perspectives

**DOI:** 10.1186/s12889-016-3312-6

**Published:** 2016-07-22

**Authors:** Jane M. Ussher, Rosie Charter, Chloe Parton, Janette Perz

**Affiliations:** Centre for Health Research, Western Sydney University, Locked Bag 1797, Penrith South DC, NSW 2751 Australia

**Keywords:** Aboriginal mothers, Constructions of motherhood, Early-intervention program, Mother-child attachment, Professional perspectives, Resilience, Good mothering, Trauma, Thematic analysis

## Abstract

**Background:**

The colonisation of Australia has been associated with traumatic consequences for Aboriginal health and wellbeing, including the breakdown of the traditional family unit and negative consequences for the mother/child relationship. Early-intervention programs have been developed to assist families to overcome disadvantage and strengthen mother/child attachment. However, there is no research examining Aboriginal women’s subjective experiences and constructions of motherhood in the context of such programs, and no research on the perceived impact of such programs, from the perspective of Aboriginal mothers and healthcare workers (HCWs), with previous research focusing on child outcomes.

**Method:**

Researchers conducted participant observation of an early intervention program for Aboriginal mothers and young children over a 6 month period, one-to-one interviews and a focus group with 10 mothers, and interviews with nine HCWs, in order to examine their perspectives on motherhood and the intervention program.

**Results:**

Thematic analysis identified 2 major themes under which subthemes were clustered. *Constructions of motherhood*: ‘The resilient mother: Coping with life trauma and social stress’ and ‘The good mother: Transformation of self through motherhood’; *Perspectives on the intervention:* ‘“Mothers come to life”: Transformation through therapy’; and ‘“I know I’m a good mum”: The need for connections, skills and time for self’.

**Conclusions:**

The mothers constructed themselves as being resilient ‘good mothers’, whilst also acknowledging their own traumatic life experiences, predominantly valuing the peer support and time-out aspects of the program. HCWs positioned the mothers as ‘traumatised’, yet also strong, and expressed the view that in order to improve mother/child attachment a therapeutic transformation is required. These results suggest that early interventions for Aboriginal mothers should acknowledge and strengthen constructions of the good and resilient mother. The differing perspectives of mothers and HCWs on the role and impact of the early intervention program reinforces the need for Aboriginal mothers to be involved in the design and implementation of services aimed at assisting their families.

## Background

The colonisation of Australia has initiated a cycle of trauma and marginalisation which has had an almost universally negative impact on the health and wellbeing of Aboriginal^1^ Australians [3, 19]. The reproductive health of Aboriginal women is a matter of particular concern for health providers and policy makers, and is related to the mental health and wellbeing of mothers and their children [22, 26]. Providing support and care in the antenatal and postnatal period has been described as a “key part of closing the gap in Indigenous perinatal outcomes” ([41], p. 1). However, there is no research examining Aboriginal women’s subjective experiences and constructions of motherhood in the context of such programs, and no research on the perceived impact of such programs, from the perspective of Aboriginal mothers and healthcare workers (HCWs), with previous research focusing on child outcomes. The aim of this study is to address this gap in the research literature.

A number of psychosocial factors and social inequities have been associated with Aboriginal maternal health outcomes [19], including general life stresses, poverty, and poor housing [4], unemployment [21], domestic violence, and childhood sexual or physical abuse [25]. A significant cause of intergenerational trauma has been the routine and forcible removal of children from their parents and culture [3, 14], culminating in the historical events referred to as the ‘stolen generations’ [47]. The most recent Australian Bureau of Statistics data indicates that the national rate of Aboriginal children in foster care is more than 10 times the rate for non-Aboriginal children [1]. Additionally, Aboriginal children are removed from their families at a younger age and spend a significantly longer time in care, in comparison to a non-Aboriginal sample [7]. Since the 1997 publication of the ‘Bringing Them Home Report’ into the Stolen Generations, the rate of Aboriginal child removal has increased by 400 % [1, 24, 47]. Disparities in child removal rates are attributed to ongoing Aboriginal ‘disadvantage’; however, some researchers and Aboriginal advocates assert that Eurocentric judgements continue to be passed on Aboriginal families and their cultural parenting practices [3, 18, 27, 48].

Dominant culture informs us of “what it means to be a mother”, as well as what behaviours, attitudes, and self-identities are appropriate for mothers ([28], p. 22). Those deemed to be ‘good mothers’ are rewarded, whereas those who deviate from dominant motherhood ideologies, the ‘bad mothers’, are sanctioned [43]. If a woman perceives that she has not achieved what it means to be a ‘good mother’ the result can be intense feelings of guilt and shame [2], which can lead to depression [32]. Many Aboriginal families have had ongoing experiences of state ‘intervention’, in some cases across multiple generations, because of perceived poor mothering practice [47]. This has led to a deep fear and suspicion related to government-run family services which can prevent families from accessing services in times of need [17] out of fear of being deemed unfit parents and having their children removed [24]. Parenting under these conditions, with the weight of generations of disadvantage and inequality, has led to the breakdown of many Aboriginal families and has been associated with compromised caregiver/child attachment [31].

The reproductive health and subjective wellbeing of Aboriginal mothers has implications for the next generation. Trauma and stress experienced in utero have been associated with behavioural problems in young children [40], which can exacerbate difficulties experienced in the mother-child relationship. The quality of a child’s early environment, and the strength of the mother-child relationship, plays an important role in the child’s psychological development [37], with consequences for adult wellbeing and relationships [27]. It has been argued that the capacity of Aboriginal and Torres Strait Islander mothers to develop a secure attachment relationship and provide such a quality environment might be challenged as a result of a number of factors, such as lack of parenting skills resulting from being removed from their family as a child [27], or ongoing life stress, which can result in depression [10].

A growing body of research suggests that if at-risk families are identified early, attachment-based interventions can lead to better outcomes for both caregiver and child [31, 45]. Such interventions aim to improve the caregiver-child relationship in order to build trust and consistency in caregiver-child attachment, as well as to facilitate changes in the caregivers attunement and reaction to their child’s needs [31]. However, there is no research examining Aboriginal women’s experiences or perspectives of such programs, beyond the early post-natal period [36]. Indeed, there is little research in Australia on Aboriginal motherhood in general, with existing research primarily of an epidemiological nature [7, 11], or measuring the success of parenting or interventions through measuring child outcomes [31, 48].

It is widely recognised that effective service delivery necessitates health professionals working with Aboriginal women developing a trusting relationship, being respectful of Aboriginal culture and practices, and being reflexive about their own perspective and its influence on their practice [5, 30]. The perspective of health professionals on the development and delivery of early intervention programs aimed at facilitating Aboriginal women’s reproductive health and mothering, and their perspectives on the mothers attending such services, can provide valuable insights into the utility and effectiveness of such programs. Conducting research in partnership can also facilitate reflection on the role of health care workers in service provision, and increases the likelihood of findings being translated into practice. A second aim of this study is therefore to examine the perspectives of health practitioners working within an early intervention program for Aboriginal mothers and their babies.

## Method

### Design

Constructions of motherhood and perspectives of an early intervention program for Aboriginal mothers were examined through participant observation of the program over a six month period, one-to-one interviews and a focus group conducted with 10 mothers, and interviews with nine health care workers (HCWs). Participant observation facilitates a close and intimate familiarity with a given group of individuals and their practices through an intensive involvement in their cultural environment, usually over an extended period of time [15]. Individual interviews provide an insight into the complexities of individual experience and constructions of motherhood, eliciting personal accounts which may not be disclosed in a group setting [23]. Focus groups give insight into shared experiences and constructions [39].

### Participants

Participants were recruited through a community organisation in an urban Australian setting. The organisation provides a range of early intervention programs targeted towards young Aboriginal families. One of these programs consists of two mothers groups designed to support Aboriginal mothers and mothers of Aboriginal children. The mothers groups each met weekly and were facilitated by trained therapists assisted by support workers. The service provides an unstructured program of discussion, baby watching, art, video making, cooking and nutrition discussion. A psychodynamic framework is employed by the organisation, with particular focus on the impact and prevention of the ongoing effects resulting from inter-generational trauma. There is a particular focus on attachment and encouraging emotional engagement in the mother-child relationship. Mothers were pre-dominantly referred to the service through a hospital-based service specifically for Aboriginal mothers. All mothers who used the hospital service offered the opportunity to attend the early intervention program, with the criteria for inclusion being that they were Aboriginal with a child aged 0 to 5, or were the mother of an Aboriginal child aged 0 to 5.

The average age of the mothers was 28 years of age, and the time that they had been involved with the service varied from 3 months to 4 years. Nine of the mothers identified as Aboriginal, and one participant was the mother of an Aboriginal child. The age of the children attending the service with their mothers ranged from one week to 5 years old. Professional staff who participated in the interviews performed a wide range of roles in relation to the groups including: group leaders, supervisors of group leaders, an Indigenous artist and cultural advisor, social work and art therapy students who were working with the group as part of placements, and other support staff who assisted with childcare and food preparation. One of the staff members was Aboriginal, the remainder from a range of Anglo-Australian and European backgrounds.

### Procedure

Mothers and HCWs were initially introduced to the research via discussions with staff and stakeholders at the organisation. Upon the participants’ agreement for the research to commence, a researcher attended each of the mothers groups for a six month period, conducting participant observation, in order to become familiar with the group and its members. Following a process of informed consent, semi-structured interviews were audio recorded and transcribed verbatim. Interviews lasted between 25 and 55 min. Where mothers indicated a preference to not be recorded, researchers produced detailed field notes documenting interview accounts from memory, a practice used in previous Aboriginal research [33]. In the interviews with the mothers, questions included experiences of motherhood, life changes since having children, and experiences of the group. Mothers were given a $25AUD gift card to reimburse them for their time. Topics covered in HCWs interviews were experiences of the group, interactions with the mothers and their children, and changes the HCWs had witnessed in the mothers, the children, and themselves. The data was collected between May 2014 and July 2015.

### Analysis

Transcripts from recorded interviews and field notes from unrecorded interviews and the focus group comprised the data for this research. Thematic analysis was used to identify and describe meaningful patterns across data [6]. The data was analysed over several stages. First, the researchers familiarised themselves with the data, through repeated reading of the integrity-checked transcripts and field notes, in order to become familiar with the “depth and breadth of the content” ([6], p. 16). An initial coding framework was the generated from the data through consultation within the research team. A line-by-line coding of the data was then conducted to collate all instances of the codes that had been identified. The organised data was then used to identify commonalities, differences and patterns across the data, with higher order themes subsequently identified. Each theme was considered, not only in the context of the individual theme itself but how it related to the overall story told within the data [20]. Two main themes, each with two sub-themes, were identified: *Constructions of motherhood*: ‘The resilient mother: Coping with early life trauma and social stress’ and ‘The good mother: Transformation of self through motherhood’; *Perspectives on the intervention:* ‘“Mothers come to life”: Transformation through therapy’; and ‘“I know I’m a good mum”: The need for connections, skills and time for self’. Table 1 summarises the themes, with examples drawn from the data. In order to maintain anonymity, accounts are identified as mothers or HCWs in the presentation of the analysis below.

**Table 1:** Thematic summary of the data

Constructions of motherhood:	Health Care Worker Accounts	Mothers’ accounts
*The resilient mother: Coping with early life trauma and social stress*	*Absence of parenting* “She is very upset and distressed in thinking about her own mother. She has always said that is never going to happen to him, referring to her own son”. *Social stress* “Many are finding the housing situation is very stressful for them and inadequate, really. One woman in the group, she’s got four children aged 8 down to one year, and she lives in a two-bedroom unit with one bathroom”. *Resilience* “Children are demanding and it’s pretty, a pretty tough gig being a mum and some of them are single, and wow that’s even more hard, so I think that the strength of some of these mums is pretty amazing stuff.”	*Absence of parenting* “I didn’t have the best childhood. And I felt towards my own child, after the way I was treated, what if I did the same to my own child? It was always in my head that it could have been better for me. I don’t want my son to have the same things happen to him. I was thinking about it all the time.” *Social stress* “Things were pretty hard for me, my partner wasn’t here when I had my baby, he was in gaol. My mother moved into my apartment to help me. Mentally it was too much to deal with.” *Resilience* “That’s not all there is to us. We want to give our kids a better life and that needs to be recognised. Sometimes I feel like people don’t want to let us move past where we’re from to something different”.
*The good mother: Transformation of self through motherhood*	“I think our mums show a lot of pride in their children because they all look very clean, they dress them well, they dress them suitably for the weather, they show that pride and care. I don’t think that’s out of fear, I think that’s just they are loving and good mums”.	“I’m really happy to be a mum, I find it probably one of the best things I have ever had to do in my life. I think I’ve become a lot more mature and a lot more confident in myself” “[being a mum has] made me a better person because before then, I really had no one because I hadn’t talked to my mum and dad, and so it was just literally me. It made me a bit more happy and it’s a great feeling being a mother”. “I have purpose now”
Perspectives on the intervention:	Health Care Worker Accounts	Mothers’ accounts
*“Mothers come to life”: Transformation through therapy*	“She was very short with her daughter and quite grumpy, whenever the daughter wanted her attention. Now I see her imitating the other mums in the group or imitating our behaviour, being very, very hands on and listening to the children. She’s now more attentive to her daughter’s needs”. “A lot of them got stronger as we spend weeks together and months… you see moments where you go, wow, that’s a bit different from the beginning…it’s just learning and growing with each other and I think the mums just, this place makes them stronger about being a mum, and the role of the mother.”	“[the group] has given me a lot more confidence because like, when I was pregnant with (child), I did have people question me, why am I pregnant and a young mum. And I was just really upset about it and I guess that’s what the group’s given me, is that confidence to just be myself and be happy that I have (child)”. “[the group] motivates me to be a good mum and everything. I don’t think what I’m doing is any different to any other mum, and I feel like all mums have to learn, no matter what age they are, like we’re not taught how to be a mum, it just comes naturally”
*“I know I’m a good mum”: The need for connections, skills and time for self.*	“It’s really powerful that their children will grow older and remember the times that they saw their mother sitting around creating art and even if it inspires one of them, that’s what we aim to do” “They care about each other. There’s a sense of camaraderie, they care about each other’s children and you know, notice the development of other people’s babies”. “I think being together, being together and a shared experience of mothering. I think that’s what groups do very well. I think it’s in a sense they’ve got a history together now of over a year, they’ve got a history with their kids and they do enjoy the milestones of each of the children, they enjoy all that with each other which is a lovely support I think.”	“When we’re doing our paintings, we talk to each other. That’s good sometimes, just to see where the other mums are at. I’m home alone with (my child) the rest of the week so it’s good to just talk about things with someone who is, you know, in the same boat. Who’s not gonna judge you if you’re having a hard time” “I like to paint, I haven’t painted for years, so it was nice to be able to relax and just go into another room where I can just paint. I don’t get time to do anything like that at home. When I come here I can just focus on that and be a bit free up, in my head, you know?” “When I get there, all I want to do is sit down, because it’s my only break to sit and talk to people. So, yeah, it’s a big socialising thing for me”.

As non-Aboriginal researchers working in an Aboriginal service it is imperative that we reflect upon our own impact and position within the research process [49]. RS and CP kept a reflexivity journal, containing field notes and observations pertaining to data collection, facilitating understanding of personal assumptions and positions of privilege that influence the way research is conducted). As a team we also reflected upon our own subject positions during the analysis, as white, middle class professional women, with only one of us being a mother (RC).

## Results

### Constructions of motherhood

#### The resilient mother: Coping with early life trauma and social stress

The construction of mothers as resilient in the face of life trauma and ongoing social stresses was dominant in the accounts of both mothers and HCWs. Many of the mothers recounted their own experience of familial breakdown, with some acknowledging that their biological mothers had been unable to raise them. The most common reason cited for this was maternal “drug” and “drinking” issues, however, one participant spoke of “issues with my mum’s partner” also being a factor. One participant described herself as “a DOCS [Australian Social Services] child”, raised in an adoptive family, whilst the other women were raised by grandmothers, grandfathers and aunts. Their experiences varied, with one woman feeling “very angry” and “struggling to fit in” with her adopted family, whereas another woman said that although it was “stressful on account of his drinking” her maternal grandfather sacrificed a lot in order to raise her and her sister and had provided her with the stability she needed, making sure “food was in the fridge… (we had) clothes on our back, a roof over our head and that we went to school every day”. HCWs also recognised the difficulties experienced by the mothers. One HCW stated that the mothers’ “own experiences of being parented, their own very early experiences aren’t good. So they don’t have a lot to sort of fall back on or to call on as mothers, you know, particularly as first-time mothers.” Another commented: “they are women who have hated their mothers often, or felt deprived or felt robbed in really psychotic transference relating to their mothers”. When asked if it is difficult to parent in instances where they did not have many positive parenting experiences of their own, the mothers offered a variety of responses. Some expressed “a lot of sadness”, “resentment” and “anger” towards their own mother’s inability to care for them as children. Other participants expressed their resignation: “it’s all in the past”, “there’s no point crying about it now”, with one participant noting that she “had learnt something from her mother” and that is “what not to do”, demonstrating resilience.

Many of the mothers spoke about traumas and stresses that they had experienced in adulthood, including difficult partner and family relationships, domestic violence and struggles with alcohol. These experiences were described as having an influence on their experiences of motherhood. As one woman said, “I didn’t really have a good relationship with [my child]’s dad” and “I think it wasn’t till my ex and I broke up that I became more positive about the pregnancy.”

For a minority of the women, their experience of trauma and greatest need for resilience was in relation to the removal of their own children. One woman described how she “didn’t get to raise” her eldest child, who was brought up by her mother. She noted, with some sarcasm, that this is “funny to (her) now” as her mother was “unable to raise (her)” for periods during her childhood due to her own struggle with alcohol. Another mother noted, “I didn’t really get the chance to be a mum” to her oldest two children, who were taken by local social services due to domestic violence. For these mothers, the removal of their children was described as “heart breaking” which brought with it “depression”, loss and grief:

The day they took my baby away from the hospital. I don’t think you can ever get over that. How can someone do that?… take a baby from someone at the hospital, when you’re still recovering from birth.

This mother described her experience as “the worst feeling in the world” and noted that it was the feeling of helplessness, that she “had no control over anything”, that she struggled with the most. There was also the added feeling of “betrayal” from institutions who “just take babies away”, and the courts, that she felt were supposed to help mothers.

The women who discussed traumatic experiences universally expressed the need to “move past” hopelessness and work towards improving things in order to care for their children, and for women who had had children removed, to be reunited with those children, demonstrating their resilience. Actions, such as “quitting drinking” and leaving abusive long-term partners, were taken. The Mothers said that the most important things were to “not give up” and to find the “right support”. The right support was described as offering “lots of options”, “trust” and “listening to what I needed”. One woman said: ‘Now, I feel more strong about it. Like, I am seeing my other kids more now and I am working really hard so I can give (my youngest) a better chance, so that things will be better for (us).” In constructing themselves as resilient, the mothers resisted being reduced to a traumatised identity. For example, when talking about challenging life experiences, one mother said,

That’s not all there is to us. We want to give our kids a better life and that needs to be recognised. Sometimes I feel like people don’t want to let us move past where we’re from to something different.

HCWs also constructed the women “tough” and “strong” with “an enormous amount of resilience” in the face of past trauma and ongoing life stresses. One example given by a HCW was of a school-age mother who was juggling the demands of her baby whilst knowing she had an essay to complete, when she was physically very tired: “it just felt like, just the strength there that was portrayed to me in the sense of ‘I can do this and I’m going to do it’ (laughs). Yeah I think of other kids at 15, that’s way beyond them”. Another HCW described the women’s resilience thus: “they make decisions, they choose to change their lives, they choose to keep their babies, they choose, they’ve got more natural skills. At least three of them are at school or at Uni”. The mothers were described as having “an expectation and a self-respect that they have a right to a life” because “life has not defeated them…and they want their kids to do better than they did”.

This sense of self-respect was achieved in the face of an external social gaze that was inherently critical and a social context that was often fraught with difficulty. One of the ongoing challenges acknowledged by both HCWs and mothers was the social stigma experienced by Aboriginal mothers. For example, one mother said:

I mean it’s just – we are all going through the same thing, and know what it’s like to be stereotyped as a typical young black mum. Like, that we’re black, we’re obviously gonna fall pregnant really young.

The possibility that “they won’t be able to be a good mum” because they were young, Aboriginal and a mother was a constant pressure that the mothers said that they felt they had to deal with. A couple of the HCWs also noted the extra scrutiny that Aboriginal women face as mothers, noting the ongoing impact that DOCS and the loss of custody of children had had on the mothers: “Aboriginal women feel under the spotlight, under a lot of scrutiny as parents.” Housing issues were also discussed by both the women and the HCWs, with this described by the mothers as a significant concern. This included small and “overcrowded housing”, unhealthy houses, lack of housing stability and difficulties dealing with housing agencies. For example, when asked what might make her experience of being a mother easier, one participant said, “Housing could fix the house. Then I won’t feel so stressed all the time. About like mould growing everywhere and things that are broken”. For others, issues such as family conflict, familial substance abuse, “lack of support” from extended family, and the stress associated with raising multiple young children were reported as major stressors. In these accounts of difficulties within the social contexts of the women’s lives, the mothers did not position themselves as traumatised. Instead, they focused on actions they were taking to make life better for themselves and their children, which demonstrated that they were not only resilient, but also good mothers.

#### The good mother: Transformation of self through motherhood

Whilst the mothers recognised the very real traumas and stresses that they had experienced, they positioned motherhood as a transformational process that motivated them to overcome these difficulties, constructing themselves as a good mother in the process. As part of the transformational experience of motherhood, there were contrasts in many of the mothers’ accounts of life before and after having children. For example, when asked about life before children, one mother spoke about “no direction”, in comparison to life after children, wherein it was stated that “I want to succeed now”, “I am more motivated”, and “I know what’s important now”. Another mother attributed life differences to “loving someone so much”, whilst many other mothers attributed these changes to wanting to offer their child “a better life” and “more opportunity” than they themselves had. As part of these accounts, the mothers spoke about motherhood as rewarding and providing purpose and meaning in their lives. This included the meaning gained through the love and bond that the mothers had with their children, as well as their enjoyment of their children. This was exemplified by one mother, who said: “I’m really happy to be a mum, I find it probably one of the best things I have ever had to do in my life.” A number of the mothers said that the changes that they had experienced personally following the birth of their children had made them a “better” or “less selfish” person. As one participant said,

like now, and just the little things, like the money that I have, I don’t buy myself anything, when all I wanted to do was buy myself things. You know I get excited about little things like buying a highchair, you know, like it’s just, you become so much less selfish.

As part of being a good mother, many of the mothers said that having children had also forced them to “grow up”. Growing up involved changes such as quitting “drinking”, “smoking” and “going out”, trying to obtain more stable housing, and distancing themselves from “bad relationships”. These changes were deemed vital in order to protect themselves and the welfare of their children. The mothers also gave accounts of the “sacrifice” attached with parenthood. One participant said “there’s a lot of sacrifice, I guess. You gotta give up things, dreams. Be responsible for someone else”. Another also acknowledged this, saying: “it is a big sacrifice too. Like, I do struggle with that a bit. I do feel like I’ve lost my identity a bit. But, you know, it’s been replaced with a lot of other things”. A number of the mothers talked about the difficulties inherent in this self-sacrifice, such as no longer seeing friends and experiencing social isolation. As one mother said, “I can’t just get up and go whenever I want, I’ve missed every single one of my friends’ twenty-firsts. You know, I’ve lost a lot of friends.” Some of the mothers also spoke about the broader challenges inherent in being a good mother, including exhaustion, temper tantrums, or the changing needs of the children. For example, one mother said, “I know I’m a good mum but I wish I could be more patient sometimes. I think when you’re a good mum, there’s a lot of love and time spent.” While these experiences were talked about as challenging, the mothers constructed these experiences as an expected part of motherhood, not particular to their identities as ‘Aboriginal mothers’, thus normalising their experiences. A number of the HCW’s also normalised the stresses of motherhood the women experienced: “children are demanding and it’s a pretty tough gig being a mum and some of them are single, and that’s even more hard, so I think that the strength of some of these mums is pretty amazing stuff”.

### Perspectives on the Intervention

#### “Mothers come to life”: Transformation through therapy

A number of HCWs also described the mothers as “great mums”, but focused on the impact of “inherited neglect and trauma” on the women’s “confidence as a parent” and the transformative power of the groups in facilitating the development of good motherhood. As one HCW told us:

They are great mums and all that and I hate to use the word, better, but maybe it’s stronger or something, some other word like empowering them, that just says that this individual is able to handle life and all the stuff that goes in people’s lives, a lot easier than before they came here.

The impact of the groups on the behaviour of children was also central in the HCW accounts. For example, a HCW described a “little boy who is so troubled, kind of disturbed” but as “the mother has become less anxious and more confident, and felt more supported” in the group, this has “changed the quality of how she is able to relate to him and that’s helped to improve his behaviour”. From the perspective of HCWs, their key role was perceived as being “facilitators” in “improving attachment” between the mothers and their children. This was described as being achieved in a number of ways, such as through “holding, noticing and observing emotions” and “interactions” between the women and their children, “connecting with culture through art”, and “breaking isolation” through “giving (the women) a great day out” as well as the “opportunity to spend time together”. Through observation, HCWs tried to identify if women were experiencing significant problems and then follow up with them. Key aims were to help the women “identify their feelings”, and receive appropriate “support to address trauma”. One HCW stated, in relation to the groups’ aims and outcomes:

The program has helped the women to just build more capacity as mothers, more maternal capacity. (To) be able to think about the kids and who they are as individuals is a really important aim.

HCWs cited the importance of “focus(ing) on the needs of the mothers” and assisting mothers in “making a connection with their children”, as illustrated in the following vignette:

One woman in particular who, when she first came to the group, the baby was only six weeks old and she was premature so the baby was just….unsupported across the mother’s lap, and the mother’s looking away. There was no eye contact, there was no proper firm holding of the baby, no support of the little head, and no emotional connection. You really could sense the deadness in the mum, and I actually was frightened for the baby…The baby wasn’t putting on weight, even though she was being fed, and we were very worried. Other health services were involved…DOCS were worried, you know, the postnatal support services were very worried. So she’s coming to the group and was seeing one of the psychotherapists here, as well. And gradually as, I guess as more trust built up between her and the therapist, she was able to tell her about how her own father had suicided when she was six months old, actually in a tree in the backyard of the family home… She told her about this, and as she was telling her, she put the baby to the breast and the baby fed for the whole session, and it was like a corner was turned like a connection was made, and the baby started to put on weight. And the two of them together started to come to life, and I’m absolutely convinced that that’s a combined effect of the support she had in the group and the input from the therapist as well. And I don’t think it’s an exaggeration, to say, because the baby could have died, you know, that really could have happened.

This account demonstrates the potential transformative power of therapeutic aspects of the groups, from the perspective of the HCWs. From an attachment perspective, this narrative draws attention to the experience of trauma, its causation, and how it may manifest within the mother/child relationship. Additionally, it illustrates how family trauma can be transmitted both interpersonally and intergenerationally [3].

When asked about challenging aspects of the groups, HCWs acknowledged reticence amongst the women to engage with elements of the program, in particular “conversations”, “talking” and “sharing”. This was put down to the notion that the women may feel “that their parenting is being scrutinised”, which in turn, elicits “shame and suspicion”. When asked why the women may feel like this, one HCW stated that: “I think it’s very hard for Aboriginal people to trust institutions and government agencies”.

#### “I know I’m a good mum”: The need for connections, skills and time for self

The mothers placed a somewhat different emphasis on the nature and function of the group and did not attribute their ‘good mothering’ to the group. When asked whether they felt they were learning anything about motherhood through their attendance at the groups, most mothers responded by saying “I know I am a good mum”. Some of the mothers talked about motherhood as something that was “natural” and was therefore something they did instinctively, as one mother said, “it can’t change.” Furthermore, some of the women positioned supportive interventions as something needed by ‘other mothers’ who were not engaging in the good mothering practices that they were. For example, when asked if the group had changed the way she felt about being a mother, one woman said:

Not really… you hear these stories about people who are taking drugs and whatnot. Yeah, I’m not going to do stuff like that. They’re just stories, nothing that mothers here are doing. So, I don’t think it’s going to change anyway.

When asked about what they got out of attending the group most mothers focused on peer social support, such as “seeing the other mums”, “each other”, “I come for my girls”:

When we’re doing our paintings, we talk to each other. That’s good sometimes, just to see where the other mums are at. I’m home alone with (my child) the rest of the week so it’s good to just talk about things with someone who is, you know, in the same boat. Who’s not gonna judge you if you’re having a hard time.

As evidenced by the above account, a number of the mothers were single and gave accounts of providing the day to day care of their children on their own. As such, the mothers group was often the only time they had in their week to relax, “have a breather” and socialise with other mothers and take part in other activities offered in the group: “I like coming to the playgroup because it gives me at least a little time to relax”; “because it’s my only break to sit and talk to people. So, yeah, like it’s a big socialising thing for me.” This camaraderie and peer support was reported to have given them greater confidence and made them feel like “better mums”:

Going to the group made me feel like a better mum ’cause at first, finding out I was pregnant at a young age, I didn’t feel too good about myself but knowing there’s other young mums out there doing the same thing as me helped.

The HCWs also acknowledged that the women were “really comfortable with each other” and that the group offered the opportunity for the mothers to “break their isolation”, “have fun together” and offer each other support:

I think the women support each other amazingly. They’re warm to each other by and large. I don’t want to guild the lily but they care about each other. There’s a sense of camaraderie, they care about each other’s children and notice the development of other people’s babies.

Other enjoyable aspects of the groups were “the kids seeing their friends”, as well as activities such as the art group, where “I can just focus on that and be a bit free up, in my head” in one woman’s account.

Each of the mothers positioned herself as active and positive in her parenting role with many of the mothers also articulating a desire to educate herself and improve as a parent. When asked what they needed to help them achieve this they cited “educational things” related to “my child’s development”, “help to understand my child’s behaviour and how to deal with it”, “story time”, “music and singing songs”. One participant suggested learning skills that “connect the group with the home” would be most beneficial, “learning different activities that I could take back with me”. When asked what she thought the groups should emphasise one mother said:

Educating mothers about how to care for their children, how to teach your kids things, especially if the kids don’t go to childcare. Teaching health things, like about brushing teeth every day, how to feed your kids, changing people’s habits, showing them different ways to parent.

At the same time, a number of the mothers did talk about how they appreciated the support that they received from the staff. For example, one mother said,

I feel like I get support from them, they’re encouraging me like I’m doing the right thing. I’m doing a good job by raising [child’s name] … just saying every week how much she’s growing, and saying that, we all, all us mothers are doing a good job raising our babies.

A couple of the mothers also spoke about the support they received from the service providers as counteracting some of the stigma that they experienced as young Aboriginal mothers.

Having the support from [HCW] and [HCW] was really good as well, like they don’t think it’s a bad thing to be a young mum, so I thought it was good to have that support.

The type of support that was appreciated by the mothers was consistent with their constructions of themselves as ‘a good mother’, valuing what the women are *doing* as mothers and acknowledging the strengths of the women. Some of the mothers also talked about their attendance at the group as a reflection of being good mothers, for example, using the group to provide their children with positive peer interactions with other children.

## Discussion

The aim of this study was twofold: to examine how a group of Aboriginal Australian mothers experience and construct motherhood and to examine how these Aboriginal mothers and their HCWs construct the impact of an early-intervention and attachment-based support group. The Aboriginal mothers who attended the groups recognised the very real traumas they had experienced, yet positioned motherhood as something that was motivating them to overcome these difficulties. They expressed a belief in their own mothering practice and agency in improving the lives of their children, identifying their own resilience in the face of day-to-day challenges of raising children with very little resources or support. They also reflected on the ramifications of their own early life trauma in terms of their own mothering. At the same time, the mothers consistently constructed themselves as ‘good mothers’ and invested significantly in this construction.

By constructing themselves as both traumatised *and* resilient/good mothers the participants in this study took on a both/and position in relation to their experiences, a practice previously described as ‘tight-rope talk’ [35]. Tight-rope talk enables the mothers to reject the binary positions of either/or, traumatised *or* resilient, in order to articulate a more complex account of their experiences which acknowledges *both* difficulties and personal strength. In this context, adopting more than one subject position as mothers is not contradictory, rather it reflects the complexity of subjective accounts neither negating nor simplifying aspects of one’s experience [46]. McKenzie-Mohr and Lafrance ([35], p. 66) describe this adoption of a “both/and” position as enabling the re-authoring of emancipatory counter-stories, which serve to challenge the oversimplification of “either/or” binaries, where women are “agent or patient”, “powerful or powerless”; or in the case of Aboriginal mothers, traumatised *or* resilient/good mothers. As Catrina Brown [9] has argued, this “both/and” position “honors women’s agency and power while not minimizing the impact of oppressive social discourses and social relations” (p. 275). These findings reflected the work of Crenshaw [13] and Collins [12], who assert that by exploring the ‘lived experiences’ of marginalised individuals we give expression to a more complex and nuanced understanding of the individuals multiple identities.

In the context of the groups, HCWs viewed motherhood as a time of vulnerability and potential. The trauma and resilience of the mothers was recognised, within a both/and dichotomy, but emphasis was placed on the role of the group in creating or boosting that resilience, thus facilitating the women becoming ‘good’ mothers. By forging a therapeutic relationship, the ‘traumatised mother’ could be healed and the bond between mother and child strengthened. From the perspective of the HCWs, the priority is for the mothers to receive therapy in order to address childhood trauma and strengthen their ability to form secure attachments with their children. The mothers may indeed have been transformed therapeutically by their attendance at the groups and derive benefit from the therapeutic process, as portrayed in the HCW accounts. However, the mothers themselves did not position the groups as functioning in this manner, focusing on peer support, time out, and enjoyment of activities, such as art. One explanation for these differing perspectives is the mothers’ potential resistance to acknowledging the need for therapeutic intervention, with its attendant implications of poor parenting practice, given their previous experiences or knowledge of state intrusion and judgement. The sense of one’s parenting being monitored, or needing ‘intervention’, is an understandable concern amongst many Aboriginal women, given the extent of state intervention and judgement both historically and currently [3]. A second explanation is simply that the mothers derived great pleasure and benefit from their connections with the other women attending the group, as is the case with any mother-baby group [29], and so this was fore-front in their minds when asked about the function of the group. A third explanation is that the mothers do not view themselves as needing therapeutic intervention, positioning this type of support as only necessary for ‘other mothers’ who are not engaging in good mothering practices, such as those who use drugs.

Given the statistics on Aboriginal removal from family it is perhaps not unexpected that these experiences were prevalent within many of the mother’s accounts. For these mothers, their experiences of loss, and the anger associated with their own mothers’ inability to care for them pervaded much of their childhoods. As demonstrated by Bromfield et al. [7] removal from troubled family situations is not always the hoped for panacea. For many of the women, being placed into multiple foster families or passed between different relatives may have further compounded their trauma and fragmented their vulnerable identities. These accounts also illustrate that the trauma surrounding these experiences can be carried well into adulthood and, for some, repeated in their own parenting, evidenced in the transgenerational cycle trauma documented in many Indigenous communities [3, 18]. Since the publication of the ‘Bringing them Home Report’ [47] there has been little exploration into the contemporary Indigenous experience of removal from family. What research there is, like much Indigenous research, is heavily epidemiological [8]. Given the increasing prevalence of Indigenous removal from family in Australia, research exploring the impact it has on families and individuals is sorely needed. Additionally, whilst Australian government policy mandates a connection with family be maintained, there has been little in the way of program development in Aboriginal communities aiming to support families and assist them in addressing the issues causing family breakdown [24].

In combination, the accounts of the mothers and HCWs illustrate constructions of motherhood operating within an early intervention program, as well as the differing strategies each adopted in terms of facilitating mothering and strengthening mother/child attachment. The mothers communicated a strengths-based approach, emphasising enhancing existing competencies and learning new skills, as well as connecting with other mothers, whilst the HCWs adopted a professional-led deficit-based approach that focused on addressing underlying trauma [42]. The mothers actively resisted being positioned solely as ‘traumatised’ as this could imply that they are ‘bad mothers’, not capable of caring for their children. The ‘bad mother’ discourse is particularly significant to Aboriginal mothers. As *women*, they are bound by dominant motherhood ideologies which dictate that being deemed a ‘bad mother’ excludes one from the ‘cult of domesticity’ that defines femininity [16] and means one has failed as a woman [2]. As Aboriginal women, to be positioned as a ‘bad mother’ creates a double bind of the intersecting marginalisations embedded within the social categories of gender and race [16]. There can be severe implications in being positioned as an Aboriginal woman who is a ‘bad mother’, the punishment being the possibility of having one’s children removed. The reality of this consequence is evident within the mother’s accounts as well as throughout modern Australian history [48].

There are a number of strengths and limitations to this study. The strengths are the use of qualitative interviews with Aboriginal women, the participant observation of the group to inform the analysis and facilitate data collection, and the inclusion of HCW perspectives. The limitations are the small sample size and collection of data from a single organisation. However, it was not the aim of this study to generalise these findings to all Aboriginal mothers or all parenting support programs for Aboriginal mothers, but rather to provide an exploratory analysis of the ways in which this group of mothers and HCWs constructed and experienced motherhood. Additionally, it is important to note that “Aboriginal mothers” is not an homogeneous category, and rather it is a collection of accounts from individuals who come from a variety of backgrounds, and who may choose to identify themselves in multiple ways and at different times, rather than be held captive by their cultural affiliation [18]. A further limitation was the intensive nature of the participant observation, which required commitment on the part of the research team and the organisation. Future researchers could explore other modes of engagement with such programs, such as attending less frequently, but at key stages of program development. Another strategy could be to involve the mothers in the development of the research questions and methodology, thus developing trust and familiarity early in the process. The use of other means of data collection, such as cultural probes, could also be considered; women taking photographs of their experience is a method used successfully in previous research with young women [34, 44]. It would also be useful to explore Aboriginal women’s experiences of other forms of supportive program. The group examined in the present study focused on mother-child attachment and encouraging emotional engagement in the mother-child relationship, through a combination of group discussion, structured activities, and one-to-one interaction. Other forms of support may also be efficacious, and may result in different experiences for both the women and the HCWs.

## Conclusion

In conclusion, the findings of this study are important insofar as they provide insight into how this group of Aboriginal mothers experience and construct motherhood and how motherhood is experienced in the context of a supportive program. Results contribute to knowledge by illustrating the multifaceted way in which these Aboriginal mothers positioned their mothering identities and how they resisted being defined solely as disadvantaged. Additionally, the study provided insight into how supportive programs are perceived by HCWs and how, in turn, they are received by those who access them. Some researchers have questioned the appropriateness of using Western-research based models of bonding and attachment with Aboriginal families [48]. This study demonstrates the potential benefits of an early intervention using an attachment model, but also demonstrated the need for HCWs to consult with Aboriginal women when developing programs intended to benefit Aboriginal families, including the need to focus on Aboriginal women’s resilience and strength, and to reinforce their sense of themselves as good mothers. The current study also illuminated an area of research that requires further research attention - contemporary experiences of Aboriginal removal from family, a significant issue as is evidenced by statistics on current Aboriginal child removal rates [1]. Additionally, it may be beneficial for future studies to interview Aboriginal women who attend different types of interventions, as well as those who do not access these services, including Aboriginal women from different backgrounds and those who are from different geographical areas. Findings of this future research may provide a more complete picture of the marginalisation experienced by Aboriginal Australians and bring us closer to bridging the gap in Indigenous health and wellbeing.

## Footnotes

1 The term Aboriginal is used throughout this paper in recognition that Aboriginal peoples are the first inhabitants of New South Wales, Australia.
